# Cognitive impairment in NMOSD—More questions than answers

**DOI:** 10.1002/brb3.1842

**Published:** 2020-10-06

**Authors:** Dominika Czarnecka, Magdalena Oset, Iwona Karlińska, Mariusz Stasiołek

**Affiliations:** ^1^ Department of Neurology Medical University of Lodz Lodz Poland

**Keywords:** cognition, cognitive dysfunction, magnetic resonance imaging, neuromyelitis optica spectrum disorder, neuropsychological tests

## Abstract

**Introduction:**

Neuromyelitis optica spectrum disorder (NMOSD) is a type of central nervous system antibody‐mediated disease which affects mainly optic nerves and spinal cord, but may also present with acute brainstem syndrome, acute diencephalic syndrome, and cerebral syndrome with typical brain lesions. One of the most disabling symptoms, diagnosed in 29%–67% of cases, is cognitive dysfunction, with such processes as memory, processing speed, executive function, attention, and verbal fluency being predominantly affected. However, description of cognition in NMOSD patients is still a relatively new area of research.

**Methods:**

A systematic MEDLINE search was performed to retrieve all studies that investigated cognitive impairment and its clinical correlates in patients with NMOSD.

**Results:**

We summarize the current knowledge on cognitive impairment profile, neuropsychological tests used to examine NMOSD patients, clinical and demographical variables affecting cognition, and magnetic resonance imaging correlates. We provide a comparison of cognitive profile of patients with multiple sclerosis and NMOSD.

**Conclusion:**

Patients with NMOSD are at significant risk of cognitive deficits. However, the knowledge of cognitive symptoms in NMOSD and potential modifying interventions is still scarce. Further accumulation of clinical data may facilitate effective therapeutic interventions.

## Graphical abstract

One of the most disabling symptoms of demyelinating diseases, diagnosed in 29%–67% of patients with NMOSD, is cognitive dysfunction, with such processes as memory, processing speed, executive function, attention, and verbal fluency being predominantly affected. However, description of cognition in NMOSD patients is still a relatively new area of research. In this review, we summarize the current knowledge on cognitive impairment profile, neuropsychological tests used to examine NMOSD patients, clinical and demographical variables affecting cognition, and magnetic resonance imaging correlates.

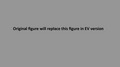

## 1 INTRODUCTION

Since the first cases of neuromyelitis optica (NMO) had been described by Eugene Devic in 1894, the understanding of pathogenesis and clinical presentations of the disease broadened considerably (Wingerchuk et al., 2015). The discovery of an association of NMO with the presence of serum antibodies against aquaporin 4 (AQP4) facilitated the diagnostic process of the disease and allowed for better differentiation between NMO and multiple sclerosis (MS) variants. In the central nervous system (CNS), the water channel AQP4, involved in the processes of osmoregulation, is highly expressed on astrocytic end‐feet (forming part of the blood–brain barrier) and ventricular ependyma (Mader, Brimberg, & Diamond, 2017; Rosales & Kister, 2016; Wingerchuk, Lennon, Pittock, Lucchinetti, & Weinshenker, 2006).⁠ Importantly, also Müller cells of the retina express this membrane protein, what can lead to alter fovea shape in AQP4‐IgG(+) patients (Motamedi et al., 2020; Yamamura & Nakashima, 2017). Moreover, AQP4 is widespread in many organs outside CNS, such as kidneys, placenta, stomach, skeletal muscle, lungs, the inner ear, lacrimal and salivary glands, and olfactory epithelial cells (Rosales & Kister, 2016).

In the years following the description of antibodies against AQP4 (AQP4‐IgG), multiple clinical observations revealed AQP4 seropositivity also in patients with clinical syndromes not fulfilling the strict NMO criteria. This leads to the introduction of the concept of NMO‐spectrum disorder (NMOSD), with core clinical characteristics encompassing optic neuritis, acute myelitis, area postrema syndrome, acute brainstem syndrome, acute diencephalic syndrome, and cerebral syndrome with typical brain lesions (Wingerchuk et al., 2015).

Recently, serum autoantibodies specific for myelin oligodendrocyte glycoprotein (MOG) have been considered as another biomarker associated with NMOSD clinical picture (Weinshenker & Wingerchuk, 2017). MOG is a protein localized on the extracellular surface of myelin sheaths and in the outer surface of the oligodendrocytes (Marignier, Cobo Calvo, & Vukusic, 2017; Michaela, Vincenza, Matteo, Marta, & Giancarlo, 2018; Narayan et al., 2018). Antibodies against MOG (MOG‐IgG) have been detected in serum of 25%–42% of AQP4‐IgG seronegative NMSOD patients (Narayan et al., 2018; Weinshenker & Wingerchuk, 2017). These data suggest an existence of at least two separate pathophysiological pathways underlying the clinical symptoms fulfilling the criteria of NMOSD—“astrocytopathy” with secondary myelin destruction in AQP4‐IgG seropositive patients versus primary “myelinopathy” in patients with serum MOG‐IgG (de Sèze, Kremer, & Collongues, 2016). Interestingly, the accumulating clinical observations of AQP4‐IgG and MOG‐IgG NMOSD cases reveal also differences in disease course and response to therapy. In consequence, exclusion of MOG‐IgG seropositive patients from the wide NMOSD category as a “MOG‐disease” spectrum has been postulated by several authors (Jurynczyk et al., 2017; Ramanathan et al., 2018; Zamvil & Slavin, 2015).⁠

Neuromyelitis optica spectrum disorder occurs all over the world and affects both men and women from 3 to 80 years old. The average age of onset is 40 years (Weinshenker & Wingerchuk, 2017).⁠ The ratio of affected women and men is 5:1 to 10:1. The prevalence of the disease differs depending on the population examined and also according to the NMO/NMOSD diagnostic criteria used in particular epidemiological studies, with the range between 0.5 and 10 per 100,000 (Patterson & Goglin, 2017). NMOSD cases are predominantly sporadic; however, few familial cases have also been described (Bruscolini et al., 2018).

The range of NMOSD’s symptoms is very wide. Optic neuritis typically causes visual loss accompanied by pain, particularly during eye movements (Patterson & Goglin, 2017; Sand, 2016). LETM often results in a complete spinal cord syndrome—paraparesis below the level of the lesion, bilateral sensory symptoms, and loss of sphincter control. Myelitis in NMOSD is also frequently accompanied by neuropathic pain, pruritus, and paroxysmal tonic spasms (Chan, 2011; Rosales & Kister, 2016). Area postrema is a structure located in the dorsomedial part of the medulla oblongata (Baghbanian, Asgari, Sahraian, & Moghadasi, 2018).⁠ An area postrema syndrome is characterized by intractable hiccups, nausea, and vomiting, and all these symptoms can occur together or separately (Han, Yang, Zhu, & Jin, 2017; Sand, 2016).⁠ Lesions of the brainstem can lead to hemiparesis, dysphagia, respiratory disturbance (Akaishi, Nakashima, Sato, Takahashi, & Fujihara, 2017)⁠⁠, oculomotor abnormalities, pruritus, hearing loss, facial palsy, trigeminal neuralgia, vertigo or vestibular ataxia, other cranial nerve abnormalities and excessive yawning. The NMOSD symptoms may result also from diencephalic dysfunction. Hypersomnia or narcolepsy is the effect of hypothalamic pathology. Diencephalic symptoms in NMSOD may also include: disturbances of temperature regulation (including hypothermia, hyperthermia, and poikilothermia), anorexia or obesity, endocrinologic abnormalities (e.g., syndrome of inappropriate secretion of antidiuretic hormone (SIADH) (Sand, 2016)), irregular menstruation, and hyperprolactinemia (Akaishi et al., 2017)⁠, diffuse anhidrosis, and altered level of consciousness, which is probably associated with thalamic lesions. Cerebral lesions can result in focal neurologic deficits, depending on the location involved. Posterior reversible encephalopathy syndrome (PRES) and hydrocephalus resulting from inflammation and occlusion of cerebral aqueduct, have also been described (Baghbanian et al., 2018; Sand, 2016). Moreover, about half to two‐thirds of patients with NMOSD with brain lesions present cognitive impairment. Cognitive processes as memory, processing speed, executive function, attention, and verbal fluency are predominantly affected (Whittam et al., 2017).⁠ Importantly, impaired learning, memory, information processing speed, and attention may occur in patients without visible brain lesions during an acute relapse (He, Chen, Zhao, & Zhou, 2011). Additionally, depression and anxiety, as well as fatigue, are frequently associated with NMOSD and have a significant impact on health‐related quality of life (HRQoL) (Whittam et al., 2017).⁠⁠ The symptoms associated with other organs, which may occur in NMOSD, encompass primary retinopathy (Motamedi et al., 2020; Yamamura & Nakashima, 2017), asymptomatic hyperCKemia and corticosteroid‐responsive myalgia, microcystic macular edema (MME), hearing loss, and hyposmia (Rosales & Kister, 2016).⁠

In this review, we summarize the currently available knowledge on the cognitive impairment in NMOSD patients.

## 2 COGNITIVE IMPAIRMENT PROFILE IN PATIENTS WITH NMOSD

Reports concerning cognitive impairment in the course of NMOSD are sparse. Nonetheless, a growing interest has been arising in this topic since NMOSD can be better differentiated from other demyelinating diseases. Recently, frequency of cognitive deficits, its characteristics, and intensity as well as associations with clinical and radiological symptoms were described.

In studies published until now, the frequency of cognitive impairment was reported at the level of 29%–67% of patients with NMOSD (Blanc et al., 2008; Chanson et al., 2011; He, Wu, et al., 2011; Hollinger et al., 2016; Kawahara et al., 2014; Kim, Kwak, Hyun, et al., 2016; Kim, Kwak, Jeong, et al., 2016; Vanotti et al., 2013; Wang et al., 2017; Zhang et al., 2015). In one of the first reports, results of neuropsychological tests were compared between groups of 30 patients with NMOSD, MS, and healthy controls (HC). Cognitive deficits were detected in 57% of patients with NMOSD, mostly affecting information processing speed, attention, verbal fluency, and executive functions. Similar results were observed in patients with MS (Blanc et al., 2008). Another author described cognitive dysfunction even in 67% of patients with NMOSD, with deficits mostly in memory, attention, and executive function (Moore et al., 2016). Nevertheless, in several studies the frequency of cognitive dysfunction was lower—between 29% and 36% (Kim, Kwak, Hyun, et al., 2016; Kim, Kwak, Jeong, et al., 2016; Zhang et al., 2015). In two reports, no difference in frequency was found between groups of patients with NMOSD and HC, but in those studies computerized tests were used to assess cognitive function and even the authors acknowledged that such tests may not be sensitive enough to detect deficits among patients with NMOSD (Hollinger et al., 2016; Kawahara et al., 2014). In summary, majority of analyses confirmed that patients with NMOSD scored significantly lower in cognitive tests than HC. Deficits in attention, memory, processing speed, verbal fluency, verbal learning, and executive function are considered to be the most frequent in this group of patients (Blanc et al., 2008; Chanson et al., 2011; He, Wu, et al., 2011; Hollinger et al., 2016; Kawahara et al., 2014; Kimiskidis et al., 2016; Vanotti et al., 2013; Wang et al., 2017; Zhang et al., 2015), as shown in Table 1.

**Table 1:** Studies on cognitive function in NMOSD patients

References	Amount of patients (AQP4‐IgG seropositive/AQP4‐IgG seronegative)	% of patients with cognitive dysfunctions	Cognitive impairment criteria	Battery of used tests or used tests	Area of cognitive dysfunctions	Additional informations
Blanc et al. (2008)	30 (17/13)	57	>2 standard deviations in any test	BccogSEP (French modified version of BRB‐N)	Memory, processing speed, verbal fluency, executive function	
Blanc et al. (2012)	28 (12/16)	54	<5th percentile in any test	BccogSEP (French modified version of BRB‐N)	Attention, memory, processing speed, executive function	Reduction of brain volume positively correlates with cognitive dysfunctions
Moore et al. (2016)	42 (30/12)	67	>2 standard deviations in any test	CVLT‐II, WAIS‐IV, SDMT, phonemic and semantic fluency tests	Attention, processing speed, verbal learning, verbal fluency, executive function	
Saji, Arakawa, et al. (2013))	14 (14/0)	57	<5th percentile in at least 3 tests	BRB‐N	Attention, memory, processing speed	
Hollinger et al. (2016)	25 (No data)	No data	No data	DANA (battery of computer tests)	Attention, memory	
He, Wu, et al. (2011))	21 (No data)	No data	No data	CVLT‐II, DST‐backward, CLOX, SDMT, PASAT	Attention, learning, memory, processing speed, executive function	All patients during acute relapse cognitive dysfunction during an acute relapse correlates with FA and MD in local regions of corpus callosum—FA is lower and MD higher than normal control subjects
He, Chen, et al. (2011))	22 (No data)	No data	No data	CVLT‐II, DST‐backward, CLOX, SDMT, PASAT	Attention, memory, processing speed	Comparison to patients with depression
Meng et al. (2017)	Not applicable	Not applicable	Not applicable	PASAT, COWAT, CVLT‐II, SDMT, WCST	Processing speed, working memory, verbal fluency, verbal learning, executive function	Meta‐analysis and systematic review
Eizaguirre et al. (2017)	Not applicable	35–67	Not applicable	Not applicable	Attention, information processing speed, verbal fluency, verbal episodic and visual memory, executive functions	Review
Fujimori et al. (2017)	12 (12/0)	Not applicable	>2 standard deviations in at least 2 tests	BRB‐N, WAIS‐III Revised, WMS‐R	Not applicable	BRB‐N overestimates cognitive dysfunction in middle‐aged NMOSD patients
Kawahara et al. (2014)	10 (No data)	No data	No data	MMSE, HDS‐R, FAB (battery of computer tests)	No cognitive impairment	
Vanotti et al. (2013)	14 (No data)	57	<5th percentile in any test	BRB‐N	Attention, memory, verbal fluency	
Kim, Kwak, Hyun, et al. (2016)); Kim, Kwak, Jeong, et al. (2016))	82 (71/11)	29	<5th percentile in at least 3 tests	SVLT, HVLT‐R, RCFT, COWAT, SDMT, PASAT DST, Stroop Color and Word tests	Attention, processing speed	
Kim, Kwak, Hyun, et al. (2016)); Kim, Kwak, Jeong, et al. (2016))	91 (82/9)	Not applicable	No data	SVLT, RCFT, COWAT, SDMT, PASAT, DST	Not applicable	Lower overall cortical thickness in NMOSD patients than in healthy controls—no correlation with neuropsychological test scores
Hyun et al. (2017)	91 (No data)	Not applicable	No data	SVLT, RCFT, COWAT, SDMT, PASAT, DST	Not applicable	Significant thalamic atrophy in NMOSD patients— no correlation with neuropsychological test scores
Kim et al. (2017)	73 (65/8)	32	<5th percentile in at least 3 tests	SVLT, HVLT‐R, RCFT, COWAT, SDMT, PASAT Digit Span, Stroop Color and Word tests	Attention, memory processing speed	White matter microstructural damage and deep gray matter (DGM) atrophy are the strongest predictors for cognitive dysfunctions in NMOSD patients
Zhang et al. (2015)	36 (25/11)	36.1	>2 standard deviations in at least 2 tests	MACFIMS, MMSE, MoCA,	Memory, processing speed	
Masuda et al. (2017)	16 (15/1)	Not applicable	No data	WAIS‐III, WMS‐R, TMT, CAT	Not applicable	Better cognitive function of NMOSD patients than patients with MS
Wang et al. (2015)	50 (32/18)	Not applicable	No data	CVLT‐ II, PASAT, SDMT, COWAT, WCST	Memory, processing speed, verbal fluency	Reduction of brain volume positively correlates with cognitive dysfunction
Liu et al. (2015)	54 (38/16)	48.2	≥1.5 standard deviations in at least 2 tests	MACFIMS	Memory, processing speed, verbal fluency, executive function	
Wang et al. (2017)	20 (11/9)	Not applicable	Not applicable	mPASAT	Not applicable	Abnormal baseline brain activity in NMOSD patients—during mPASAT performing

Abbreviations: BRB‐N, Brief Repeatable Battery of Neuropsychological Tests; CAT, Clinical Assessment for Attention; CLOX, executive clock drawing task; COWAT, Controlled Oral Word Association Test; CVLT‐II, California Verbal Learning Test‐Second Edition; DANA, Defense Automated Neurobehavioral Assessment; DST‐backward, backward Digit Span Test; FAB, Verbal Fluency and Frontal Assessment Battery; HDS‐R, Hasegawa Dementia Scale‐Revised; HVLT‐R, Hopkins Verbal Learning Test‐Revised; MACFIMS, Minimal Assessment of Cognitive Function in Multiple Sclerosis; MMSE, Mini‐Mental State Examination; MoCA, Montreal Cognitive Assessment; PASAT, Paced Auditory Serial Addition Test; RCFT, Rey Complex Figure Test; SDMT, Symbol Digit Modality Test; SVLT, Seoul Verbal Learning Test; TMT, Trail Making Test; WAIS, Wechsler Adult Intelligence Scale; WCST, Wisconsin Card Sorting Test; WMS‐R, Wechsler Memory Scale‐Revised.

The high variation in the reported prevalence of cognitive impairment in NMOSD may result from several reasons. One of the most important issues is the heterogeneity of examined groups of patients with NMOSD in terms of ethnic background, antibody status, type of treatment, and occurrence of disease relapse (Liu et al., 2015; Moore et al., 2016; Vanotti et al., 2013). The majority of studies were conducted on small samples—10 to 12 patients, mostly of Asian origin. It is also relevant that assessment of cognitive function was performed with different neuropsychological tests. Use of ununified batteries of tests makes it difficult to compare results between studies (Fujimori et al., 2017; Kawahara et al., 2014). Thus far, no battery of tests was created specifically to examine cognitive function in NMOSD. Usually, the cognitive performance of patients with NMOSD was evaluated according to current recommendations for the evaluation of MS patients. Popular batteries of tests such as Brief Repeatable Battery of Neuropsychological Tests (BRB‐N) and Minimal Assessment of Cognitive Function in Multiple Sclerosis (MACFIMS) (see Table 2) as well as individual tests allowing to assess particular domains were used. In this manner, the Symbol Digit Modalities Test (SDMT) is used to assess visuospatial processing speed, Paced Auditory Serial Addition Test (PASAT) for auditory processing speed, California Verbal Learning Test‐Second Edition (CVLT‐II) for verbal learning, Controlled Oral Word Association Test (COWAT) for verbal fluency, and Wisconsin Card Sorting Test (WCST), Frontal Assessment Battery (FAB), and the Stroop Color and Word Test (SCWT) for executive functions (Blanc et al., 2008; Fujimori et al., 2017; Saji, Nishizawa, & Kawachi, 2013; Vanotti et al., 2013). Moreover, in various studies different criteria were used to identify patients with cognitive impairment. Some authors confirmed the diagnosis when 2 tests were failed, others used less strict criterion—3 tests had to be done improperly. The matter is even more complicated due to the heterogeneity of the definition of a test failure. In some of the studies, scoring <1 standard deviation (*SD*) was demanded, while in the others, 2 *SD* were chosen as a threshold (Kim, Kwak, Hyun, et al., 2016; Kim, Kwak, Jeong, et al., 2016; Moore et al., 2016; Vanotti et al., 2013; Zhang et al., 2015). It is of great importance that popular tests screening for dementia such as MMSE or some computerized tests are proved to be not useful in evaluation of cognitive impairment in NMOSD patients (Kawahara et al., 2014; Zhang et al., 2015).

**Table 2:** Batteries of tests used in evaluation of NMOSD patients

Cognitive functions	BRB‐N
Verbal memory	Selective Reminding Test (SRT)
Visuospatial memory	10/36 Spatial Recall Test (SPART)
Attention, processing speed	Symbol Digit Modalities Test (SDMT)
Attention, processing speed	Paced Auditory Serial Addition Test (PASAT)
Verbal fluency	Word List Generation (WLG)
Spatial processing	
Executive function	

Abbreviations: BRB‐N, Brief Repeatable Battery of Neuropsychological Tests; MACFIMS, Minimal Assessment of Cognitive Function in Multiple Sclerosis.

## 3 CLINICAL AND DEMOGRAPHICAL VARIABLES AFFECTING COGNITION AND QUALITY OF LIFE OF NMOSD PATIENTS

Several studies investigated whether such clinical and demographical factors as age, level of education, level of neurological disability, mood disorders, and length of the disease may influence the spectrum and intensity of cognitive dysfunction and quality of life (QoL) in NMOSD patients.

A few studies considered associations between duration of the disease and severity of cognitive deficits (Hollinger et al., 2016; Vanotti et al., 2013). It is possible that, similarly as in MS, cognitive dysfunction may occur in a very early stage of the disease and progress with its duration; however, there is still not enough follow‐up research in this field. Nevertheless, only in one of the published reports no cognitive impairment was detected in a group of patients with the disease duration <3 years (Kim, Kwak, Hyun, et al., 2016; Kim, Kwak, Jeong, et al., 2016). The clinical importance of these observations seems to be supported by the fact that increasing age may have an additional negative impact on the results of neuropsychological evaluation, especially in tests measuring information processing speed, attention, and short‐term memory (SDMT, PASAT) (Saji, Nishizawa, et al., 2013).

To date, no unequivocal connection between level of physical disability (in all of the studies measured by EDSS) and results of neuropsychological tests was found. In several reports, higher EDSS scores were significantly associated with poor cognitive performance (Blanc et al., 2012; Moore et al., 2016; Saji, Arakawa, et al., 2013; Vanotti et al., 2013). Significant correlation between SDMT and EDSS scores was described in one of the studies (Blanc et al., 2008). However, no associations of this type were observed by other groups (Liu et al., 2015; Vanotti et al., 2013; Zhang et al., 2015). Importantly, based on the available data, cognitive impairment in NMOSD seems to be independent of other symptoms and may already affect patients with low level of physical disability. Patients with severe physical dysfunction may be still fully cognitively capable. It is suggested that cognitive impairment is also independent of disease exacerbation and seems to be permanent and progressive (Oertel, Schließeit, Brandt, & Paul, 2019).

It is important that treatment of acute relapse, which usually includes high‐dose corticosteroids, has also been associated with a range of behavioral side effects such as depression, mania and psychosis and a disruptive effect on cognition, including difficulties with concentration, memory, working memory, and executive functioning skills. Additionally, a meta‐analysis encompassing 26 studies suggested that length of the treatment had an impact on diverse cognitive domains, and acute therapy had negative effect on executive function and very long‐term memory but positive effect on expressive language. On the other hand, short and long duration of steroid therapy had negative impact on recent memory. These effects appear to be dose‐dependent, but reversible after discontinuation of treatment (Prado & Crowe, 2019). Thus far, impact of immunosuppression and immunomodulation on cognitive function in NMOSD patients has not been profoundly investigated and the potential influence of drugs such as azathioprine, mycophenolate, and other long‐term treatments on cognition is yet to be described in this disease.

One of the commonly occurring symptoms in NMOSD is pain—it affects even up to 86% of patients and is usually described as mild in intensity (Asseyer et al., 2018). Interestingly, for the characterization of disease‐related pain, MOG‐Ig‐positive NMOSD patients chose in the McGill Pain Questionnaire descriptions such as “throbbing,” “pushing,” “annoying” in contrast to AQP4‐Ig positive patients who preferred words “flashing,” “pricking,” “tingling” (Asseyer et al., 2018). Pain in patients with NMOSD correlates with emotional affect and depression scores and is an independent predictor of physical aspects of QoL (Asseyer et al., 2018). Although, in NMOSD pain is usually described as mild in intensity (Asseyer et al., 2018), in one of the studies pain was more frequent and more severe in a group of patients with NMOSD than in patients with MS (83.8% vs. 47%) (Kanamori et al., 2011). This finding is of special meaning in the light of the possible correlation between severity of pain and the extent of cognitive deficits (Saji, Arakawa, et al., 2013).

Interestingly, it was also observed that sleep may be a particularly important factor for cognitive performance in patients with NMOSD as it was found that they were more vulnerable to the cognitively impairing effects of sleep deprivation than healthy individuals (Hollinger et al., 2016).

Since AQP4 was suggested to play a role in synaptic plasticity (Scharfman & Binder, 2013), AQP4‐IgG status was also examined in the context of cognitive deficits (Saji, Arakawa, et al., 2013). However, thus far no relationship between presence of AQP4‐IgG and cognitive parameters was observed in patients with NMOSD (Liu et al., 2015; Moore et al., 2016; Zhang et al., 2015).

Another important factor related to cognition in patients with NMOSD is a level of education. Many reports show a positive correlation between the level of education and scores in neuropsychological test in NMOSD patients (Kim, Kwak, Hyun, et al., 2016; Kim, Kwak, Jeong, et al., 2016; Vanotti et al., 2013; Zhang et al., 2015). Therefore, higher level of education may be a protective factor for cognitive deficits in this group.

Scarce data are available regarding the impact of cognitive impairment on employment status and social life of NMOSD patients. It was reported that rates of unemployment in this patient group are high (up to 65%) (Beekman et al., 2019). However, no data are available regarding the possible influence of cognitive dysfunction on vocational status. Although patients with NMOSD confirm that the disease has a high impact on their social life and relationships with family and friends (Beekman et al., 2019), studies considering cognitive impairment in this area are still lacking.

## 4 DEPRESSION AS A SIGNIFICANT FACTOR MODIFYING COGNITION

All of the studies investigating frequency of depression among patients with NMOSD concluded that severity of depression is higher in NMOSD patients than in the general population. According to various estimates, depression affects 42%–58% of patients with NMOSD (Blanc et al., 2008; He, Chen, et al., 2011; Vanotti et al., 2013). However, only half of those with moderate or severe depression are treated and receiving antidepressants (Chavarro et al., 2016). In one of the reports, which included groups of patients with NMOSD, MS, and HC (42 patients in each group) examined with a well‐validated structural psychiatric interview, the lifetime prevalence of depression was found to be high in both NMOSD and MS patients (46% and 34%, respectively), but even up to 27% of patients with NMOSD and only 5% with MS had experienced recurrent depression and suicidality (Moore et al., 2016). Moreover, among patients with NMOSD higher intensity of depressive symptoms was associated with more pronounced neurological disability and poor cognition (Kawahara et al., 2014; Moore et al., 2016). Nevertheless, only several other studies investigated possible connections of depression and cognitive impairment in patients with NMOSD. Depression has been associated with lower scores in tests evaluating information processing speed (He, Chen, et al., 2011) or attention (Vanotti et al., 2013). However, the results of available studies are incoherent (Blanc et al., 2008; Kawahara et al., 2014; Saji, Arakawa, et al., 2013). Apathy has been suggested as yet another condition increasing cognitive deficits in NMOSD patients (Kawahara et al., 2014). Thus, it should be emphasized that mood disorders and cognitive impairment tend to increase each other's prevalence in NMOSD patients, but interconnections are not yet clearly explained.

In NMOSD, similarly as in MS, fatigue is an important factor influencing cognition. It can be reported as one of the initial symptoms by ca. 35% of patients (Beekman et al., 2019) and affect even up to 70% of them throughout the course of the disease (Seok et al., 2017). Together with depression fatigue influences cognition and QoL of the most patients with NMOSD (Hollinger et al., 2016; Kim, Kwak, Hyun, et al., 2016; Kim, Kwak, Jeong, et al., 2016). Patients with more severe fatigue reported also worse quality of sleep and higher depression rates (Seok et al., 2017). Intensity of fatigue correlated significantly with results of SDMT in patients assessed up to 6 weeks after disease relapse (He, Chen, et al., 2011). Importantly, both depression and fatigue had a significant influence on routine daily activities of patients with NMOSD. Noteworthy, ability to work was defined in this group of patients as one of the most important determinants of QoL together with pain and impact of the disease on career (Beekman et al., 2019).

In one of the studies, patients with NMOSD scored lower than HC in all subtests of HRQOL scale, but exceptionally high correlation was found between fatigue, depression, and decrease of physical and mental composite scores of HRQOL. Additionally, fatigue was observed especially among patients with more severe physical disability (Chanson et al., 2011). Although the analysis encompassed only patients with relatively low accumulation of neurological deficits (mean EDSS = 3.5) in spite of a long duration of disease (mean 9.3 years), their QoL was significantly lower than in HC. There was no difference found in QoL between patients with NMOSD and SM (Chanson et al., 2011).

## 5 COGNITIVE IMPAIRMENT—NMOSD VERSUS MS

The differences between MS and NMOSD neuropathology may suggest an existence of distinct patterns of cognitive decline. However, such an assumption finds no support in numerous published studies (Blanc et al., 2008; Liu et al., 2015; Moore et al., 2016; Saji, Arakawa, et al., 2013; Vanotti et al., 2013). In a recent analysis, no significant differences between NMOSD and MS patients were demonstrated in SDMT, CVLT‐II, or COWAT scores. Although, both patients with MS and those with NMOSD performed poorer than HC (Meng et al., 2017). Only in a few reports cognitive impairment was more frequent in NMOSD than in MS (Saji, Arakawa, et al., 2013). In the study employing BRB‐N neuropsychological battery applied in patients with MS, NMOSD, and HC, cognitive impairment (failure in ≥3 tests) was detected in 57% of patients with NMOSD and 47% of patients with MS (Saji, Arakawa, et al., 2013). Patients with NMOSD scored significantly worse than HC in tests assessing memory, attention, and information processing speed (Saji, Arakawa, et al., 2013). On the contrary, in another study cognitive impairment (defined as a score lower than the fifth percentile compared with HC in at least three domains) was present in 29% of NMOSD patients and 50% of MS patients, in spite of longer duration of the disease and higher level of disability in NMOSD group. Additionally, patients with MS had greater cognitive deficits, mostly in such domains as verbal learning and visual memory, while in NMOSD patients information processing speed and attention were more affected (Kim, Kwak, Hyun, et al., 2016; Kim, Kwak, Jeong, et al., 2016).

Significant limitations of these comparative studies should be acknowledged and the results should be interpreted with caution due to the small study groups and distinct cutoffs of neuropsychological tests adopted as indicative of cognitive impairment. Moreover, usage of batteries of tests implemented typically in MS evokes methodological concerns in NMOSD. The tests are not standardized for age and other variables (Fujimori et al., 2017), whereas patients with NMOSD are on average ca. 10 years older than those with MS (Fujimori et al., 2017; Kawahara et al., 2014). The study of Fujimori and colleagues advocates these concerns by comparing the application of standardized neuropsychological tests (Wechsler Memory Scale, WAIS‐R) and BRN‐B (widely used in MS) in the assessment of cognitive function of 14 NMOSD and 14 MS patients. Cognitive impairment was detected by WAIS‐R in 1 patient with NMOSD and 5 with MS, while BRN‐B indicated cognitive deficits in 5 patients with NMOSD and 4 with MS (Fujimori et al., 2017). Another study showed that tests commonly used for screening for cognitive deficits such as MMSE are generally more useful in patients with MS than NMOSD (Kawahara et al., 2014). In summary, variety of neuropsychological tests applied in reported studies, small groups of patients, substantial demographic (age, level of education) and clinical (occurrence of relapse, stage of the disease, pharmacological treatment, comorbidities) inhomogeneity have to be regarded as crucial confounders, which make the comparison of cognitive deficits between NMOSD and MS very complex and hard to interpret.

## 6 RADIOLOGICAL CORRELATES OF COGNITIVE IMPAIRMENT IN NMOSD

The most common brain abnormalities in NMOSD are nonspecific small lesions in subcortical and deep white matter, which probably accumulate with the duration of the disease (Kim et al., 2015). Some characteristic findings in magnetic resonance imaging (MRI) of the CNS were incorporated in diagnostic criteria for NMOSD without AQP4‐IgG or unknown AQP4‐IgG status (Wingerchuk et al., 2015). Those include lesions corresponding with acute optic neuritis (optic nerve MRI with T2‐hyperintense lesion or T1‐weighted gadolinium‐enhancing lesion extending over 1/2 optic nerve length or involving optic chiasm), acute myelitis (intramedullary MRI lesion extending over 3 contiguous segments (LETM) or 3 contiguous segments of focal spinal cord atrophy in patients with history compatible with acute myelitis), dorsal medulla/area postrema lesions and periependymal brainstem lesions (Wingerchuk et al., 2015). Additionally, different types of nonspecific CNS lesions can be found in MRI of NMOSD patients. Lesions in corpus callosum are observed in less than a half of the patients, but may involve the whole thickness of splenium or form an extensive longitudinal lesions contiguous to pyramidal tract (Kim et al., 2015). In brain hemispheres, the lesions are often tumefactive or have long spindle‐like or radial shape following white matter tracts. These brain lesions are more commonly found in AQP4‐IgG seropositive than seronegative patients (Matsushita et al., 2010). However, results concerning ovoid MS‐like lesions are equivocal, with some of the studies reporting higher prevalence of such lesions in CNS imaging of AQP4‐IgG(−) (Downer et al., 2012; Fan et al., 2017; Matsushita et al., 2010) and others in AQP4‐IgG(+) patients (Cabrera‐Gomez & Kister, 2012). Importantly, focal cortical lesions frequently observed in patients with MS are not characteristic for patients with NMOSD (Calabrese et al., 2012; Sinnecker et al., 2012). Thus far, only 10 patients with cortical lesions were described in the literature, of whom 6 were AQP4‐IgG(+) (information about remaining cases was not provided) and 90% had specific cortical symptoms. In all of these cases, cortical lesions were accompanied by leptomeningeal involvement, which may be relevant in mechanisms of entrance for antibodies (Sun, Sun, Huang, Wu, & Yu, 2019).

Although CNS regions known as engaged in cognitive processes may be affected in NMOSD, the studies investigating imaging correlates of cognitive impairment are rather scarce and inconclusive. Thus far, no association has been described between the extent of cognitive deficits and focal CNS lesions, neither noncharacteristic nor MS‐like (Kim et al., 2017; Liu et al., 2015; Zhang et al., 2015).

In recent years, new area of research opened together with the implementation of volumetric MRI protocols enabling measurements of whole brain and/or regional atrophy. However, the extent and clinical relevance of CNS atrophic changes in NMOSD remain the topic of ongoing scientific debate and the available data seem to differ considerably depending on analyzed populations (Finke et al., 2016; Matthews et al., 2015). Atrophy of gray and white matter has been repetitively studied in NMOSD patients and compared with MS (Blanc et al., 2012; Liu et al., 2015; Wang et al., 2015). White matter volume was shown to be reduced in patients with MS in comparison to AQP4‐IgG(+) patients (Fan et al., 2017), whereas decreased thalamic and hippocampal volumes were reported in AQP4‐IgG(−) and MS patients, as compared to the HC (Fan et al., 2017).

The correlation of cognitive function with CNS atrophy in NMOSD is most probably very complex and demanding to analyze. In one of the studies, reduction of multiple volumetric parameters including volume of gray matter, hippocampus, nucleus accumbens, putamen, caudate, and thalamus was demonstrated in patients with NMOSD in comparison to HC. However, only the level of atrophy of hippocampus was significantly higher in cognitively impaired than cognitively preserved patients (Liu et al., 2015). In another study, cognitively impaired NMOSD patients were characterized by significantly more pronounced atrophy of the thalamus than patients without cognitive deficits and HC (Hyun et al., 2017). Association of cognitive impairment with right thalamic deep gray matter atrophy and regional atrophy of gray matter in the prefrontal cortices was demonstrated in yet another study (Wang et al., 2015). Cortical thinning has been reported in NMOSD patients in multiple areas such as premotor cortex, dorsolateral prefrontal cortex, superior prefrontal gyrus, middle temporal gyrus, and part of parieto‐occipital region. However, no significant correlations between cortical thickness in those regions and cognitive function of NMOSD patients were demonstrated (Kim, Kwak, Hyun, et al., 2016; Kim, Kwak, Jeong, et al., 2016; Pittock et al., 2006). White matter atrophy was also indicated as an important variable influencing cognitive parameters (Blanc et al., 2012; Kim et al., 2017; Liu et al., 2015). Global brain and focal white matter atrophy were found in a group of NMOSD patients with cognitive impairment as compared to the patients without cognitive deficits. The reduction of focal white matter volume, particularly in brainstem, corticospinal tracts, corpus callosum, and superior and inferior longitudinal fascicles, was correlated with cognitive impairment in NMOSD patients (Blanc et al., 2012). Interestingly, focal volume of the specific brain structures was correlated positively with the performance in neuropsychological tests assessing particular cognitive modalities such as working memory, information processing speed, attention, and fluency (Blanc et al., 2012).

As brain abnormalities in NMOSD patients seem to be present also in the normal‐appearing brain tissue, more advanced techniques were used to investigate the white matter damage in connection to cognition. One of the studies investigated the white matter network, based on a model of white matter connectivity established using diffusion tensor imaging and graph theory. Brain regions were considered as nodes linked by lines symbolizing white matter connections which in healthy brain are highly integrated. Performance of NMOSD patients in tests of attention, working memory and processing speed, visuospatial processing and executive function, was attributed to dysfunctions in white matter networks. The total strength of white matter networks was positively associated with PASAT3 scores, whereas reduced efficiency of the left calcarine, cuneus, and right precuneus and the reduced regional efficiency of the left calcarine were associated with decreased performance on Digit Symbol Coding Test (Cho et al., 2018).

Recently, functional connectivity density (FCD) which is a graph theory enabling to quantify spontaneous neural activity was explored in AQP4‐IgG(+) patients with NMOSD after ON. It showed significant alterations in several brain regions, such as reduced short‐range FCD in the dorsal pathway, which may stand behind impoverished vision in NMOSD and increased FCD in the left anterior cingulum and paracingulate gyri and left superior frontal orbital gyrus, which may be a compensatory effect after the damage as these regions become more active in the case of visual impairment. Interestingly, a positive association between the frequency of ON and the long‐range FCD values in the left superior frontal orbital gyrus was found in the NMOSD group suggesting an interregional functional connectivity recovery (Wang et al., 2020). Moreover, there is an assumption that structure and function of the afferent visual pathway are associated not only with vision but also with higher order cognitive processes, what is supported by a recent study which shows that functional connectivity of the primary visual network was higher in NMOSD patients than in HC and positively correlated with the results of battery of neuropsychological tests (BRB‐N) in patients, meanwhile negatively in HC, suggesting that increased connectivity is maladaptive and indicating less efficient neural network (Chavarro et al., 2020).

Also other novel MRI techniques have been employed to investigate the structural background of cognitive dysfunction in NMOSD. In diffusion tensor‐weighted imaging, significant correlation was observed between cognitive impairment and normal‐appearing white matter lesions found in the corpus callosum, anterior cingulate gyrus, and medial frontal gyrus (Eizaguirre, Alonso, Vanotti, & Garcea, 2017). Furthermore, changes in fractional anisotropy (FA) and mean axial, and radial diffusivity of normal‐appearing white matter were connected with cognitive dysfunction in NMOSD (Kim et al., 2017; Liu et al., 2015). These observations seem to suggest that NMOSD patients without lesions detectable in conventional brain MRI may have microscopic structural abnormalities in white matter, negatively influencing cognitive performance (Kim et al., 2017). Most importantly, microstructural lesions in the white matter are suggested as one of the strongest predictors of cognitive impairment in NMOSD (He, Wu, et al., 2011; Kim et al., 2017).

There are few studies available which assessed the brain activity of NMOSD patients with functional MRI (fMRI). Interestingly, reduced baseline brain activity reflected in fMRI was visualized in several areas, such as the precuneus and posterior cingulate cortex—brain regions implied to have an important role in visuospatial skills, episodic memory, and self‐processing operations. The decreased activity was also found in the lingual gyrus which is considered to be responsible for dreaming, visual processing, and visual memory (Liu et al., 2011). On the other hand, widespread abnormal increase of cerebral activation in bilateral superior temporal gyri, left inferior frontal gyrus, right cingulate gyrus, right inferior parietal gyrus, left insula, and cerebellum of patients with NMOSD performing mPASAT was found in comparison to HC. This may suggest that NMOSD patients recruit more regions of the brain to solve complicated tasks (Wang et al., 2017).

## 7 CONCLUSIONS

The majority of studies suggest that patients with NMOSD are at significant risk of cognitive deficits, affecting predominantly memory, attention, and information processing speed. Level of education, fatigue, and depression may potentially influence cognitive performance in NMOSD, and consequently patients’ ability to work, social relationships, and QoL. Unfortunately, the knowledge of cognitive symptoms in NMOSD and potential modifying interventions is still scarce. This situation may be improved by further accumulation of clinical data, optimally based on statistically representative studies performed with the use of unified batteries of neuropsychological tests and aimed to identify cognitive impairment as early as possible, which in turn may facilitate the work on effective therapeutic interventions.

## CONFLICT OF INTEREST

All authors declare no conflict of interest.

## AUTHOR CONTRIBUTIONS

Dominika Czarnecka has made substantial contributions to conception and design, searching the database and creating the manuscript. Magdalena Oset has made substantial contributions to conception and design, searching the database and creating the manuscript. She also has been involved in drafting the manuscript. Iwona Karlińska has made contributions to conception and creating the manuscript, revised it critically for important intellectual content. Mariusz Stasiołek has made substantial contributions to conception, design and creating the manuscript, revised it critically for important intellectual content and given final approval of the version to be published.

### Peer Review

The peer review history for this article is available at https://publons.com/publon/10.1002/brb3.1842.
